# Nutritional Composition and Bioactive Profiles of Farmed and Wild Watermeal (*Wolffia globosa*)

**DOI:** 10.3390/foods14101832

**Published:** 2025-05-21

**Authors:** Nidthaya Seephua, Parinya Boonarsa, Hua Li, Pornpisanu Thammapat, Sirithon Siriamornpun

**Affiliations:** 1 Department of Food Technology and Nutrition, Faculty of Technology, Mahasarakham University, Kantarawichai, Maha Sarakham 44150, Thailand; 66010853001@msu.ac.th (N.S.); 65010853004@msu.ac.th (P.B.); 2 Research Unit of Thai Food Innovation, Department of Food Technology and Nutrition, Mahasarakham University, Kantarawichai, Maha Sarakham 44150, Thailand; 3 Department of Cuisine and Nutrition, Yangzhou University, Yangzhou 225127, China; lihua216@yzu.edu.cn; 4 Key Laboratory of Chinese Cuisine Intangible Cultural Heritage Technology Inheritance, Ministry of Culture and Tourism, Yangzhou 225127, China; 5 Food Technology Program, Faculty of Agricultural Technology, Rajabhat Mahasarakham University, Maha Sarakham 44000, Thailand; thammapat.p@gmail.com

**Keywords:** alternative protein, chlorophyll, phenolic acid, phytosterol, amino acid, fatty acid

## Abstract

This study assessed the nutritional composition, bioactive compounds, phytosterol content, amino acids, and fatty acid profiles of watermeal cultivated under farm conditions (WF1) and harvested from natural environments (WF2 and WF3). WF1 exhibited the highest levels of protein (22.7%), dietary fiber (16.5%), total phenolic content (3.9 mg GAE/g DW), and total flavonoid content (5.0 mg QE/g DW). Chlorophyll and *β*-glucan contents were comparable across all samples. WF1 also showed the highest total amino acid content, while WF2 had the highest lysine and tryptophan levels. Although essential amino acid profiles were slightly below WHO/FAO/UNU reference values, watermeal remains a promising complementary plant protein source. Fatty acid analysis revealed a consistently high α-linolenic acid content (30%) across all samples, highlighting its value as a natural source of omega-3 fatty acids. Minor differences in amino acid and fatty acid profiles suggest an influence of environmental conditions. The most pronounced difference between the farmed and naturally sourced samples was observed in phytosterol content, which was highest in the farmed sample. Overall, despite variations in cultivation sources, watermeal consistently exhibits a rich nutritional profile, reinforcing its potential as a sustainable, nutrient-rich biomaterial for functional food applications.

## 1. Introduction

Watermeal (*Wolffia globosa* (Roxb.) Hartog and Plas), commonly referred to as watermeal, is a duckweed species renowned for its rich nutritional profile and ecological sustainability. Recognized as one of the world’s tiniest flowering plants [1], watermeal has been a traditional food source in Thailand and several neighboring countries for centuries [2]. Its exceptionally fast growth and efficient biomass yield make it a strong candidate for supporting global food systems and enhancing food security [3]. The plant’s potential has spurred considerable commercial interest, with the duckweed protein market projected to reach USD 72.7 million in 2024 [4]. Under optimized cultivation conditions, watermeal can yield between 10 and 30 tons of dry biomass per hectare annually [5]. Watermeal is rich in protein, dietary fiber, essential amino acids, and various bioactive compounds [1]. Watermeal is being increasingly explored for use in functional foods and as a sustainable plant-based protein source [6].

The nutritional composition of watermeal is not only diverse but also influenced by environmental factors. Studies have shown that variables such as water quality, sunlight exposure, and nutrient availability significantly affect its chemical profile [7]. Under optimal conditions, watermeal can contain over 41% protein, alongside carbohydrates, including resistant starch and dietary fibers, polyunsaturated fatty acids, and phenolic compounds with antioxidant properties [8]. Recent investigations have explored its use as a fortifying agent in food products, such as snacks [9], where the addition of watermeal has been shown to enhance both protein content and functional properties, including antioxidant activity [10]. Environmental factors also play a critical role in determining the levels of essential nutrients such as proteins, carbohydrates, lipids, vitamins, and minerals. For example, temperature fluctuations can affect enzymatic activities and metabolic pathways, while soil fertility and water quality influence nutrient uptake and bioavailability. In addition, atmospheric carbon dioxide levels impact photosynthesis and biomass accumulation [11]. In Thailand, there is growing interest in the cultivation of watermeal, supported by government initiatives and policymaker engagement. Although the consumption of watermeal has a long tradition in various local culinary practices, scientific information comparing the nutritional quality of farmed versus naturally sourced watermeal remains scarce. Particularly, there is limited data on how different growing conditions affect micronutrient levels and bioactive compound profiles in Thailand, despite the increasing trend in watermeal farming as an emerging alternative protein source.

In light of this, the current study sought to investigate the chemical makeup of watermeal that was grown on farms and collected from natural settings, with a focus on analyzing how growth conditions affected nutritional quality. Macronutrients, phytosterol concentration, bioactive compounds, and the compositions of amino and fatty acids were among the important characteristics examined. The results offer important information to help with the creation of nutrient-rich, sustainable food sources that are in line with international food security and environmental sustainability objectives, as well as to support the formulation of nutritional supplements.

## 2. Materials and Methods

### 2.1. Materials and Chemicals

The watermeal (*Wolffia globosa*) samples used in this study were categorized as follows: WF1 was cultivated under certified Good Agricultural Practice (GAP) standards using a nutrient solution (formulation SUT−NS 5), as detailed in Table 1. WF2 and WF3 were harvested from natural water sources in Nakhon Ratchasima province, Thailand (14.8882° N, 102.2548° E). All samples were collected between October 2024 and November 2024. Following collection, the materials were crushed into a fine powder using a laboratory grinder, dried for 24 h at 60 °C in a hot air oven, and then stored at −20 °C until additional analysis. The supplier of all chemicals was Sigma-Aldrich Co. (St. Louis, MO, USA). HPLC–grade (≥90% purity) standards were met for bioactive compounds, antioxidants, phytosterols, phenolic acids, flavonoids, amino acids, and fatty acids.

### 2.2. Proximate Composition Analysis

Moisture content was determined using the hot air oven method (925.10), and lipid content was analyzed via Soxhlet extraction (920.39), following the procedure described by AOAC [12]. Ash content was measured according to 923.03, and protein content was determined using the Kjeldahl method (979.09). Carbohydrate content was calculated by difference. The results were expressed as g/100 g of dried weight (DW).

### 2.3. Measuring Chlorophyll Content

A modification of this process was made from Seephua et al. [13]. The watermeal sample was treated with 80% (*v*/*v*) acetone to remove the chlorophyll. To decolorize the sample, 0.1 g of the dried sample was combined with the solvent and left in the dark. After that, measurements of absorbance were made at 645 and 663 nm. The findings were reported in mg/100 g DW.

### 2.4. Determination of β-Glucans

According to Taesuk et al. [14], the amount of *β*-glucans in watermeal was measured using a *β*-glucans test kit (Megazyme, Bray Co., Wicklow, Ireland). Three separate analyses were conducted. The findings were reported in mg/100 g DW.

### 2.5. Determination of Total Phenolic Content (TPC)

TPC was determined by using the colorimetric method with the Folin–Ciocalteu reagent. The protocol used is based on that described by Siriamornpun et al. [15]. An aliquot (0.3 mL) of the extract was mixed with 2.25 mL of 10% Folin–Ciocalteu reagent and left for 5 min. Then, 2.25 mL of 6% Na_2_CO_3_ was added, and the mixture was incubated at room temperature for 90 min. Absorbance was measured at 725 nm. Results were expressed as mg gallic acid equivalents (GAE) per gram of dry weight (DW).

### 2.6. Determination of Total Flavonoid Content (TFC)

The total flavonoid content was determined using the colorimetric method described by Wanyo et al. [16]. The reaction mixture consisted of 500 µL of extract and 2.25 mL of distilled water, followed by the addition of 150 µL of 5% sodium nitrite (NaNO_2_) solution. After a 6 min interval, 300 µL of 10% aluminum chloride hexahydrate (AlCl_3_·6H_2_O) solution was added and left to react for another 5 min. Subsequently, 1.0 mL of 1M sodium hydroxide (NaOH) was added, and the solution was thoroughly vortexed. Absorbance was measured at 510 nm using a UV spectrophotometer. Results were expressed as mg of quercetin equivalents per g of dry sample (mg QE/g DW).

### 2.7. Determination of 2,2-Diphenyl-picryl-hydrazyl (DPPH) Radical Scavenging Ability

The antioxidant activity of watermeal, following the method described by Butsat and Siriamornpun [17]. Briefly, 100 µL of the extract was added to 1.9 mL of freshly prepared 0.1 mM DPPH• solution. The mixture was then kept at room temperature in the dark for 30 min. To initiate the reaction, 500 μL of the extract was combined with 4.5 mL of a 0.1 mmol/L DPPH methanolic solution prepared fresh. The absorbance was detected at 517 nm using a UV spectrophotometer. The standard curve illustrated the free radical scavenging activity of vitamin C at varying concentrations, with the results reported as mg of vitamin C per g (mg vitamin C/g DW).

### 2.8. Determination of Ferric Reducing Antioxidant Power (FRAP)

The FRAP values of watermeal were determined using the method described by Li et al. [18]. In brief, the FRAP reagent was prepared by mixing 100 mL of acetate buffer (0.3 mol/L, pH 3.6), 10 mL of TPTZ solution (2,4,6-tris (2-pyridyl)-s-triazine) dissolved in 40 mmol/L HCl, 10 mL of 20 mmol/L FeCl_3_, and 120 mL of distilled water and prewarmed to 37 °C. Subsequently, 60 μL of the extract and 180 μL of distilled water were combined with 1.8 mL of the FRAP working solution and incubated at 37 °C for 4 min. Absorbance was immediately recorded at 593 nm. FRAP values were reported as mg of FeSO_4_ equivalents per g (mg FeSO_4_/g DW).

### 2.9. Determination of Phenolic Acid and Flavonoids by HPLC

Extraction of phenolic acids and flavonoids was carried out according to the procedure outlined by Chumroenphat et al. [19]. These compounds were subsequently identified and quantified using high–performance liquid chromatography (HPLC) (Series 20, Shimadzu, Kyoto, Japan). The operating conditions were as follows: column temperature 38 °C, injection volume 20 μL, and detection was carried out using a UV–visible detector, with wavelengths set at 280 nm for hydroxybenzoic acids, 320 nm for hydroxycinnamic acids, and 370 nm for flavonoids. The phenolic compounds in the extracts were recognized by matching their retention times and UV spectra with those of known reference standards.

### 2.10. Extraction and Determination of Phytosterols

The extraction of phytosterols followed a modified method of Thammapat et al. [20]. Samples (2 g) were saponified under nitrogen with potassium hydroxide, sodium chloride, ethanol, and ethanolic pyrogallol as an antioxidant. 5α-Cholestane (≥97.0% purity) in heptane (0.1% *w*/*v*) was added as an internal standard. The mixture was heated at 70 °C for 45 min, cooled, and extracted with n–hexane/ethyl acetate (9:1 *v*/*v*). The organic layer was evaporated, and the residue was derivatized with BSTFA:TMCS and pyridine at 60 °C for 30 min. The final extract was dissolved in heptane for the Thermo Scientific ISQ 7610 Single Quadrupole GC–MS system (Waltham, MA, USA). Separation was performed on a capillary column with helium as the carrier gas. The injection was in split mode at 280 °C. The column temperature was programmed from 60 °C to 280 °C. The quantitative analysis was based on corrected peak area ratios relative to the internal standard’s peak area.

### 2.11. Determination of Amino Acid Composition

The hydrolysate sample amino acid content was determined using the Chumroenphat et al. [19] technique with 0.05 M HCl in ethanol. A 0.22 μm nylon membrane was then used to filter the supernatant. LC/MS/MS (LCMS-8030 triple quadrupole mass spectrometer, Shimadzu, Kyoto, Japan) was performed using an HPLC system and electrospray ionization on a 2.1 mm × 150 mm, 3 μm InertSustain^®^ C18 column. The following formula from Yi et al. [21] was used to determine the essential amino acid index (EAAI). The findings are presented in terms of dry weight (g/100 g DW).

### 2.12. Determination of Fatty Acid Composition

#### 2.12.1. Lipid Extraction

Using a chloroform–methanol combination (2:1, *v*/*v*) containing 10 mg/L BHT and 0.1 mg/mL C17:0 (≥98% purity) as an internal reference, lipids were extracted from 5 g of powdered material. The mixture was filtered and mixed with 15 mL of 0.9% NaCl for phase separation after standing in a fume hood for the entire night. After being gathered and evaporated, the lower phase was adjusted to 10 mL.

#### 2.12.2. Fatty Acid Analysis

The whole lipid extracts were transesterified using a methanol solution of H_2_SO_4_ (0.9 M) to produce fatty-acid methyl esters (FAMEs). A GC system (GC-2014, Shimadzu, Tokyo, Japan) with a fused silica column (30 m × 0.25 mm, 25 μm; DB-Wax, J & W Scientific, Folsom, CA, USA) and a flame ionization detector was utilized to quantitatively analyze the resultant FAMEs. The carrier gas used was nitrogen. The temperature of the oven was set to range between 150 °C and 230 °C [1]. The following formula was used to determine the content of fatty acids:

$$\mathrm{Fatty}\;\mathrm{acid}\;\mathrm{composition}\;(\%)=\frac{area\;under\;each\;peak}{total\;area\;of\;all\;fatty\;acids\;in\;the\;chromatogram}\times 100$$

### 2.13. Data Analysis

The results of three independent experiments are shown as the mean ($\overset{-}{x}$) ± standard deviation (SD). A one-way ANOVA and Duncan’s multiple range test were used to analyze the data. IBM SPSS Statistics software, version 17.0 (Chicago, IL, USA), was used to determine statistical significance at *p* < 0.05. By using Origin 2022 software version 9.95 (OriginLab Corporation, Northampton, MA, USA), all data were log-transformed for principal component analysis (PCA) to equalize variance and avoid the principal components being disproportionately impacted by factors with higher variation.

## 3. Results and Discussion

### 3.1. Physical Appearance

**Figure 1:**
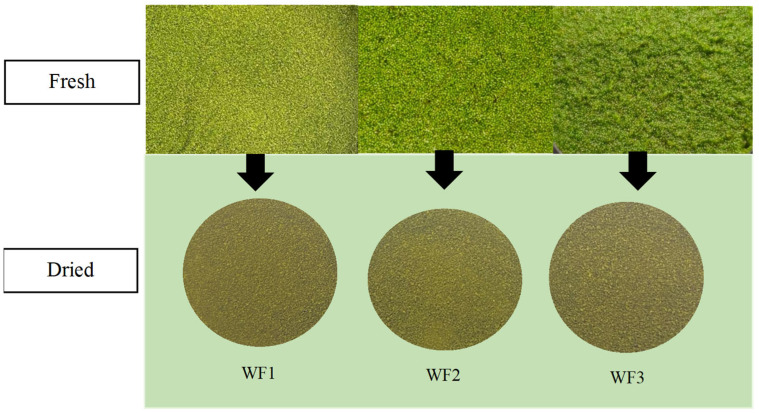
The appearance of fresh and dry watermeal from different growing sites. WF1 = watermeal cultivated under farm conditions; WF2 and WF3 = watermeal harvested from natural environments.

The appearance of fresh and dry watermeal from different growing sites. As shown in Figure 1, fresh watermeal (*W. globosa*) samples WF1 (cultivated) and WF2 and WF3 (wild-sourced) displayed visibly distinct coloration. WF2 appeared to have the most intense green hue, followed by WF3 and WF1. However, once the samples were dried, these visual differences became negligible, and all three dried samples appeared similar in color. To objectively assess pigment content, chlorophyll analysis was conducted exclusively on the dried samples. The results revealed no statistically significant differences in total chlorophyll content among the three sample groups (*p* > 0.05), suggesting that the observed visual differences in the fresh state did not correspond to actual variations in chlorophyll concentration. These findings support previous studies indicating that visual assessment alone may not be a reliable indicator of pigment content, especially after post-harvest processing such as drying [22,23]. The lack of variation in total chlorophyll content among dried samples implies that drying can act as a standardization method to unify the quality of watermeal from various sources, which is beneficial in food product development. This finding aligns with research by Hu et al. [24], who found that visual color measurement readings are limited in dried samples and should be complemented by chemical analyses for accuracy.

### 3.2. Proximate Composition

Proximate composition analysis of watermeal from different growth sites (Table 2) showed significant variations in macronutrient content. WF1 had the highest protein (22.74 g/100 g DW) and ash (7.84 g/100 g DW) levels, whereas WF2 recorded the highest lipid (4.08 g/100 g DW) and carbohydrate (52.44 g/100 g DW) contents. Dietary fiber was most abundant in WF1 (16.53 g/100 g DW), followed by WF2 (16.09 g/100 g DW) and WF3 (15.76 g/100 g DW) [1,9,25,26]. These findings suggest that environmental factors significantly influence the nutritional composition of watermeal, potentially impacting its suitability for various food and nutritional applications [27]. The farm-cultivated watermeal (WF1) received an optimized nutrient solution containing ammonium (NH_4_^+^) and nitrate (NO_3_^−^) (Table 1), which are key nitrogen sources vital for amino acid and protein biosynthesis. This optimized nutrient environment likely contributed to the elevated protein content observed in WF1 as compared to the naturally sourced WF2 and WF3, where nutrient conditions were uncontrolled. Nitrogen is essential not only for amino acid synthesis but also for chlorophyll production and cellular metabolism, all of which are critical for rapid growth and biomass accumulation. Supporting these findings, Yongyod and Kamolrat [4] investigated the effects of varying nitrogen (N) and phosphorus (P) ratios on Wolffia globosa and reported that a higher nitrogen supply, particularly when coupled with a balanced N:P ratio (especially 8:1), significantly enhanced biomass productivity and increased crude protein content. Their study demonstrated that optimal N and P concentrations stimulated growth and improved nutritional quality, with protein content increasing up to 34% in dry weight under nitrogen-enriched conditions. Conversely, nitrogen-limited treatments resulted in slower growth and lower nutritional profiles.

These results affirm that nitrogen availability is a determining factor for protein biosynthesis in watermeal and that nutrient management strategies can directly influence its nutritional value. Consequently, the significantly higher protein and ash content observed in WF1 can be attributed to the controlled, nitrogen-rich cultivation conditions. Further investigation is warranted to assess how nutrient-induced variations in composition affect the functional food potential and digestibility of watermeal [8].

### 3.3. Chlorophyll, β-Glucans, and Bioactive Compounds

**Figure 2:**
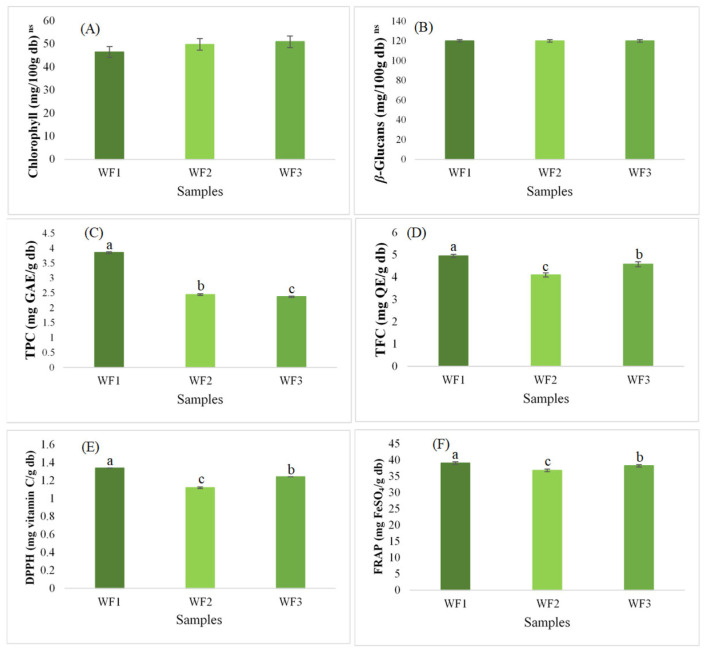
Total chlorophyll, *β*-glucans content, and bioactive compound analysis of watermeal from different growing sites: (**A**) total chlorophyll; (**B**) *β*-glucans content; (**C**) total phenolic content (TPC); (**D**) total flavonoid content (TFC); (**E**) DPPH radical scavenging activity; and (**F**) ferric reducing antioxidant power (FRAP). The column and error bar represent the mean and standard deviation (*n* = 3), respectively. Values with different letters are considered significantly different (*p* < 0.05). ns is not significantly different at *p* > 0.05. WF1 = watermeal cultivated under farm conditions; WF2 and WF3 = watermeal harvested from natural environments.

Chlorophyll, *β*-glucans, and other bioactive compounds play significant roles in enhancing the nutritional value and health benefits of food. Figure 2 presents the analysis of chlorophyll, *β*-glucans, and bioactive compounds of watermeal from different growing sites. Chlorophyll content showed no significant differences among the samples, with values ranging from 46.47 to 50.87 mg/100 g DW. This indicates that chlorophyll accumulation is relatively stable across different growth sites, likely due to its primary role in photosynthesis, which is regulated by genetic and environmental interactions [28]. Similarly, *β*-glucans content remained stable across all samples (120 mg/100 g DW), suggesting that these structural polysaccharides are less influenced by environmental variation [29]. In contrast, the bioactive compound analysis of watermeal from different growth sites (WF1, WF2, and WF3) revealed significant variations in total phenolic content (TPC), total flavonoid content (TFC), and antioxidant activities. WF1 exhibited the highest TPC (3.85 mg GAE/g DW), similar to findings from [1,10], followed by WF2 (2.44 mg GAE/g DW) and WF3 (2.37 mg GAE/g DW), indicating that the phenolic composition varies by location. Phenolic compounds are known for their antioxidant properties, and their levels can be influenced by environmental factors such as light exposure, nutrient availability, and stress conditions [30,31]. A similar trend was observed for TFC, with WF1 showing the highest value (4.96 mg QE/g DW), suggesting that environmental factors may influence flavonoid accumulation. Flavonoids contribute to plant defense mechanisms and antioxidant capacity, making them important for health-promoting effects [32]. Antioxidant activity, measured through DPPH and FRAP assays, also differed significantly. WF1 exhibited the highest DPPH scavenging activity (1.34 mg vitamin C/g DW) and FRAP value (39.12 mg FeSO_4_/g DW), suggesting that it has the strongest antioxidant potential among the three samples. This could be attributed to its higher phenolic and flavonoid content, which are known to contribute to free radical scavenging and reducing power [33,34]. Overall, these results suggest that the bioactive profile of watermeal is influenced by its growing environment, particularly for phenolics, flavonoids, and antioxidant activity. This highlights the importance of environmental factors in optimizing the nutritional quality of watermeal for potential functional food applications. Further studies on cultivation conditions and their impact on bioactive compounds could help enhance the functional properties of watermeal-based food products [3].

### 3.4. Phenolic Acids and Flavonoids Contents

Phenolic acids and flavonoids are two major groups of polyphenols found in plant-based foods [35]. The analysis of phenolic acids and flavonoids in watermeal from different growth sites (WF1, WF2, and WF3) revealed significant variations in specific compounds, while some remained unchanged (Table 3). Among the detected phenolic acids, protocatechuic acid was the most abundant, with WF2 showing the highest content (172.49 µg/g DW), followed by WF1 (170.90 µg/g DW) and WF3 (166.94 µg/g DW). WF1 (86.20 µg/g DW) and WF3 (83.14 µg/g DW) had substantially greater vanillic acid levels than WF2 (78.90 µg/g DW). Additionally, WF1 had greater levels of *p*-coumaric acid (59.80 µg/g DW) than WF2 (55.36 µg/g DW) and WF3 (56.57 µg/g DW), which might be related to its antioxidant potential. However, there were no appreciable variations in gallic acid, caffeic acid, syringic acid, sinapic acid, or cinnamic acid amongst the three groups. Remarkably, neither vanillin nor p-hydroxybenzoic acid was found in any of the samples, indicating that these substances might be absent or present in trace amounts in the watermeal from these areas. Total phenolic acid content was highest in WF1 (756.12 µg/g DW), followed by WF3 (684.52 µg/g DW) and WF2 (646.81 µg/g DW), indicating that location-based environmental factors, such as soil composition and light exposure, might influence phenolic accumulation [30,34]. Among flavonoids, quercetin and apigenin were the most abundant across all samples, with no significant differences. WF1 exhibited the highest rutin content (89.73 µg/g DW), significantly higher than WF2 (81.30 µg/g DW) and WF3 (82.50 µg/g DW). Rutin, a well-known flavonoid with antioxidant and anti-inflammatory properties, has been reported to be influenced by plant growing conditions [32]. Catechin was not detected in any of the samples, which aligns with findings in other aquatic plants where catechin levels are highly variable based on species and growing conditions [36]. Kaempferol content showed minor variations, with WF2 having a slightly higher level (42.46 µg/g DW), though the differences were not statistically significant. Total flavonoid content followed a similar pattern as phenolic acids, with WF1 (503.79 µg/g DW) exhibiting the highest levels, followed by WF3 (482.21 µg/g DW) and WF2 (473.08 µg/g DW). Since flavonoids play a crucial role in antioxidant defense, these findings suggest that watermeal from WF1 may possess higher antioxidant potential compared to the other regions [33].

### 3.5. Phytosterols Content

Phytosterols in plant-derived foods play significant physiological roles, including tumor growth inhibition, immune stimulation, and anti-inflammatory, antioxidant, and antidiabetic properties [37]. The phytosterol content of watermeal samples from three different growing sites (WF1, WF2, and WF3) is presented in Table 4 and the GC chromatogram in Figure S1. The results indicate significant variations in the phytosterol content among the samples. While campesterol was not detected in WF2, it was found in WF1 (122.70 µg/100 g DW) and WF3 (212.45 µg/100 g DW), with WF3 having considerably higher levels (*p* < 0.05). Similarly, stigmasterol was substantially greater in WF3 (212.90 µg/100 g DW) and nonexistent in WF2 but present in WF1 (144.45 µg/100 g DW). All samples included β-sitosterol, the most prevalent phytosterol; however, WF3 had the highest content (625.08 µg/100 g DW), followed by WF1 (487.41 µg/100 g DW) and WF2 (101.96 µg/100 g DW), with significant differences between them (*p* < 0.05). Only in WF1 were cycloartenol and brassicasterol found, with quantities of 43.74 µg/100 g DW and 29.88 µg/100 g DW, respectively, whereas in WF2 and WF3, they were not found.

These findings demonstrate that the phytosterol profile of watermeal is strongly influenced by the growing environment. WF1, cultivated under controlled conditions with a protein-optimized nutrient solution, exhibited the greatest diversity of phytosterols, suggesting a shift in metabolic priorities toward protein synthesis rather than sterol biosynthesis. In contrast, WF3 showed the highest levels of stigmasterol and campesterol, compounds known for their cholesterol-lowering effects [38], while WF2 also had elevated sterol content. These two samples were collected from natural environments where variable nutrient availability, light intensity, and ecological stressors likely stimulated phytosterol accumulation as part of the plant’s adaptive response [39]. Previous studies support these observations, indicating that environmental stress, such as low nitrogen availability or fluctuating light exposure, can enhance sterol biosynthesis, contributing to membrane stabilization or stress defense [40]. Additionally, Appenroth et al. [25] reported significant sterol variation in *Lemna minor* depending on cultivation conditions, which aligns with our results and further emphasizes the role of environmental factors in regulating phytosterol biosynthesis in aquatic plants.

### 3.6. Amino Acid Compositions

Amino acids in plant foods are crucial for dietary management, especially for individuals with inherited amino acid disorders, and they play a significant role in human nutrition and plant physiology. The amino acid profiles of watermeal obtained from three different sources showed significant variation in both essential (EAAs) and non-essential amino acids (NEAAs) (Table 5) and LC/MS/MS chromatograms in Figures S2–S4. Total amino acid (ƩAAs) content was highest in WF1 (15.77 g/100 g DW), followed by WF2 (15.08 g/100 g DW), and lowest in WF3 (14.01 g/100 g DW), indicating that environmental and cultivation conditions may influence protein quality and quantity. Among the essential amino acids, valine, isoleucine, leucine, and phenylalanine were present in relatively high amounts, while lysine and threonine were comparatively lower. WF1 and WF2 contained significantly higher total EAAs (8.26 and 8.25 g/100 g DW, respectively) than WF3 (7.59 g/100 g DW). WF2 exhibited the highest concentrations of tryptophan and lysine, two amino acids that are frequently deficient in plant-based proteins. All three samples, however, had lower values for the majority of essential amino acids when compared to the WHO/FAO/UNU [41] reference values for adult requirements. This means that although watermeal offers a variety of amino acids, other protein sources may need to be added in order to maintain a balanced diet. Non-essential amino acids (NEAAs), including alanine, tyrosine, and glutamic acid, are most abundant in WF1. Compared to farm-grown samples, WF1 exhibited a noticeably greater proline level, which may have been a result of responses to environmental stress. Watermeal has a wide range of amino acids and the potential to be used as a supplemental plant protein source, even if its EAA levels fell short of WHO/FAO/UNU requirements for adults. Variations across samples imply that the content of amino acids is influenced by growth circumstances. Overall, the amino acid composition of watermeal demonstrates its potential as a functional protein source, especially considering the balanced EAAs-to-NEAAs ratio and the presence of conditionally essential amino acids like arginine and tyrosine. The differences observed among samples underscore the influence of growing conditions, including water quality, nutrient availability, and environmental stressors, on protein biosynthesis in aquatic plants [42]. Although more research on protein digestibility and amino acid bioavailability is necessary, these results indicate the feasibility of watermeal as a sustainable and nutritionally significant ingredient.

### 3.7. Fatty Acid Compositions

**Figure 3:**
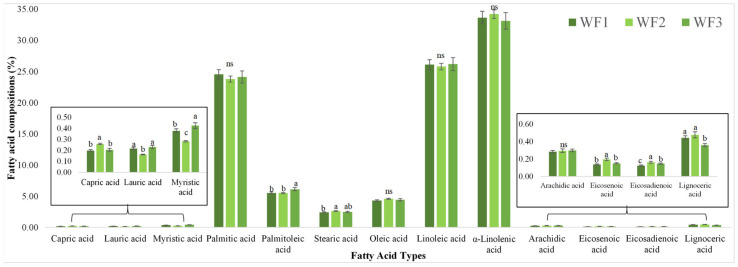
Fatty acid composition of watermeal from different growing sites. The column and error bar represent the mean and standard deviation (*n* = 3), respectively. Values with different letters are considered as significantly different (*p* < 0.05). ns is not significantly different at *p* > 0.05. WF1 = watermeal cultivated under farm conditions; WF2 and WF3 = watermeal harvested from natural environments.

Dietary fatty acids are essential for human health since they affect a number of physiological processes and the likelihood of disease [43]. The fatty acid profiles of watermeal obtained from three different sources revealed significant differences in several saturated and unsaturated fatty acids (Figure 3) and GC-MS chromatograms in Figure S5. Palmitic acid was the most prevalent saturated fatty acid in all samples, with a range of 24.5% to 25.5% and no statistically significant difference (*p* > 0.05). There were comparatively small amounts of other saturated fatty acids, such as stearic, lauric, myristic, and capric acids. In particular, WF3 had a significantly larger myristic acid content than WF1 and WF2 (*p* < 0.05), while WF2 had the lowest stearic acid level. WF3 had the largest concentration of palmitoleic acid, a monounsaturated fatty acid, although levels varied greatly between samples. However, there was no discernible difference in oleic acid between sources. All samples had high levels of polyunsaturated fatty acids (PUFAs), especially linoleic acid and α-linolenic acid. About 30% of all fatty acids were α-linolenic acid, the most prevalent PUFA. There was no discernible difference between the groups (*p* > 0.05). The nutritional potential of watermeal as a plant-based source of omega-3 fatty acids is indicated by its high α-linolenic acid concentration. The regulation of inflammation, brain development, cognition, and retinal health all depend on these critical fatty acids [44].

Overall, the fatty acid profiles suggest that watermeal possesses a beneficial lipid composition, particularly due to its high PUFA content. The variation among samples may be attributed to differences in cultivation conditions, water sources, or ecological environments, reflecting the impact of both natural and agricultural settings on the biochemical composition of this aquatic plant [42]. These findings support the potential application of *W. globosa* as a functional ingredient in health-oriented food products.

### 3.8. Principal Component Analysis

**Figure 4:**
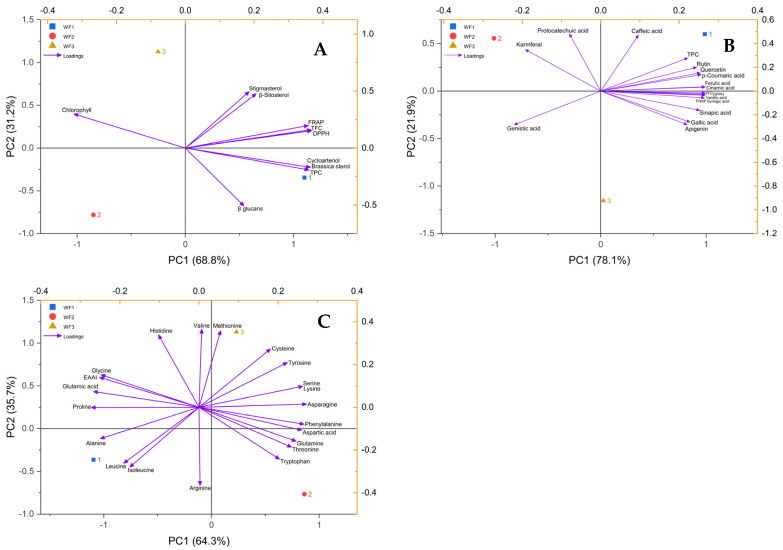
Principal component analysis (PCA) of antioxidant and phytosterol (**A**), antioxidant and phenolic and flavonoid (**B**), and amino acid composition (**C**). WF1 = watermeal cultivated under farm conditions; WF2 and WF3 = watermeal harvested from natural environments.

Principal component analysis (PCA) was conducted to evaluate the variation in bioactive compounds, antioxidant properties, phytosterols, and amino acid profiles among three different *Wolffia globosa* samples (WF1, WF2, and WF3). The three biplots together provide a comprehensive overview of the compositional differences across sample origins (Figure 4). In the first PCA plot (Figure 4A), which explains 100% of the variance (PC1: 78.1%, PC2: 21.9%), WF1 was clearly separated from WF2 and WF3 along PC1, correlating strongly with total phenolic content (TPC), total flavonoid content (TFC), antioxidant capacity (FRAP), and a suite of phenolic acids and flavonoids such as quercetin, rutin, *p*-coumaric acid, and ferulic acid. This suggests that WF1 possesses a more potent antioxidant and polyphenolic profile. In contrast, WF2 aligned with variables such as protocatechuic acid and kaempferol, indicating a different phenolic composition, while WF3 showed minimal association with most vectors, implying moderate or mixed phenolic characteristics.

The second PCA plot (PC1: 68.8%, PC2: 31.2%) focused on antioxidant activity and phytosterol contents (Figure 4B). WF1 was again strongly associated with high levels of TPC, FRAP, DPPH activity, and sterols such as cycloartenol and brassicasterol. WF2, located in the opposite quadrant, was associated more with *β*-glucans and had a negative relationship with antioxidant variables, suggesting a distinct compositional profile. WF3 was positioned closer to chlorophyll and sterols like stigmasterol and β-sitosterol, indicating a moderate phytosterol and pigment content but lower antioxidant activity compared to WF1.

The third PCA plot, which captured 100% of the variance (PC1: 64.3%, PC2: 35.7%), analyzed the amino acid profiles and essential amino acid index (EAAI) (Figure 4C). WF1 correlated strongly with branched-chain amino acids (leucine and isoleucine), alanine, and EAAI, suggesting high protein quality. WF2 was positioned opposite to these variables and instead associated with glutamine, threonine, tryptophan, and aspartic acid, highlighting differences in amino acid composition. WF3 was aligned with methionine and valine, suggesting relatively higher levels of these essential amino acids. Overall, WF1 consistently exhibited superior profiles in terms of antioxidant compounds, phytosterols, and protein quality, making it the most nutritionally and functionally rich sample. The clear distinction between WF1, WF2, and WF3 in each of the three PCA plots highlights how civilization or environmental origin affects *W. globosa*’s biochemical makeup.

## 4. Conclusions

This study demonstrates that environmental and cultivation conditions significantly influence the nutritional and biochemical profiles of watermeal. Despite variations in composition, all samples maintained high levels of valuable nutrients, particularly protein, bioactive compounds, and α-linolenic acid, supporting the potential of watermeal as a sustainable, nutrient-rich food ingredient. Differences in amino acid, fatty acid, and phytosterol profiles further highlight the role of growth environment in shaping functional properties. These insights provide a foundation for optimizing cultivation strategies to enhance nutritional quality. Future research should explore methods to improve nutrient bioavailability, investigate processing techniques to retain or enhance functional compounds, and evaluate the performance of watermeal in various food formulations such as protein-enriched products, plant-based beverages, or functional supplements. Additionally, sensory evaluation, consumer acceptance studies, and assessments of large-scale production feasibility will be critical for its successful integration into the food industry.

## Figures and Tables

**Table 1:** Nutrient solution (SUT–NS 5) of watermeal from the GAP farm standards.

Nutrient Solution	Concentration (ppm)
Nitrogen (N)	268.59
NH^4+^	30.75
NO_3_	237.84
Phosphorus (P)	30.04
Potassium (K)	290.00
Calcium (Ca)	113.56
Magnesium (Mg)	30.00
Sulfur (S)	40.00
Iron (Fe)	2.40
Boron (B)	0.51
Manganese (Mn)	1.63
Zinc (Zn)	0.44
Copper (Cu)	0.125

**Table 2:** Proximate composition of watermeal from different growing sites.

Proximate Composition (g/100 g DW)	WF1	WF2	WF3
Moisture	4.25 ± 0.24 ^a^	3.73 ± 0.7 ^c^	4.06 ± 0.67 ^b^
Protein	22.74 ± 0.66 ^a^	20.55 ± 0.43 ^b^	20.85 ± 0.58 ^b^
Lipid	3.23 ± 0.32 ^b^	4.08 ± 0.24 ^a^	3.43 ± 0.14 ^b^
Ash	7.84 ± 0.83 ^a^	6.83 ± 0.35 ^b^	6.76 ± 0.50 ^b^
Fiber ^ns^	16.53 ± 0.72	16.09 ± 0.12	15.76 ± 0.09
Carbohydrate	50.73 ± 1.03 ^b^	52.44 ± 0.26 ^a^	51.11 ± 0.62 ^ab^

The mean ± SD (*n* = 3) is used to express values. Within the same row in the parameter, means with various letters (a, b, and c) differ significantly at *p* < 0.05. ns is not significantly different at *p* > 0.05. WF1 = watermeal cultivated under farm conditions; WF2 and WF3 = watermeal harvested from natural environments.

**Table 3:** Phenolic acids and flavonoids of watermeal from different growing sites.

Parameter (µg/g DW)	WF1	WF2	WF3
Phenolic acid content			
Gallic acid ^ns^	173.04 ± 4.24	169.86 ± 3.02	173.20 ± 1.22
Protocatechuic acid	170.90 ± 0.25 ^a^	172.49 ± 2.67 ^a^	166.94 ± 1.79 ^b^
*p*-Hydroxybenzoic acid	ND	ND	ND
Vanillic acid	86.20 ± 2.07 ^a^	78.90 ± 1.25 ^b^	83.14 ± 2.08 ^a^
Caffeic acid ^ns^	13.69 ± 1.89	13.28 ± 1.27	12.62 ± 0.44
Syringic acid ^ns^	10.71 ± 1.95	9.61 ± 0.49	10.24 ± 0.46
Vanillin	ND	ND	ND
*p*-Coumaric acid	59.80 ± 2.16 ^a^	55.36 ± 0.92 ^b^	56.57 ± 0.92 ^b^
Ferulic acid	22.63 ± 2.52 ^ab^	18.02 ± 1.88 ^b^	20.15 ± 1.82 ^ab^
Sinapic acid ^ns^	137.05 ± 2.34	129.26 ± 3.74	135.60 ± 6.42
Cinamic acid ^ns^	61.38 ± 3.40	57.04 ± 1.35	59.02 ± 3.26
Genistic acid	20.72 ± 0.35 ^b^	26.29 ± 0.17 ^a^	26.64 ± 0.17 ^a^
Total	756.12 ± 2.22 ^a^	646.81 ± 1.68 ^c^	684.52 ± 2.16 ^b^
Flavonoid content			
Rutin	89.73 ± 1.62 ^a^	81.30 ± 0.10 ^b^	82.53 ± 5.30 ^b^
Catechin	ND	ND	ND
Quercetin ^ns^	195.36 ± 18.95	176.77 ± 5.63	181.30 ± 5.39
Apigenin ^ns^	179.20 ± 26.31	172.55 ± 9.15	179.98 ± 8.74
Karmferal ^ns^	39.50 ± 3.90	42.46 ± 1.41	38.40 ± 3.45
Total	503.79 ± 14.19	473.08 ± 15.23	482.21 ± 5.72

The mean ± SD (*n* = 3) is used to express values. Within the same row in the parameter, means with various letters (a, b, and c) differ significantly at *p* < 0.05. ns is not significantly different at *p* > 0.05. ND: not determined. WF1 = watermeal cultivated under farm conditions; WF2 and WF3 = watermeal harvested from natural environments.

**Table 4:** Phytosterol content of watermeal from different growing sites.

Phytosterols (µg/100 g DW)	WF1	WF2	WF3
Campesterol	122.70 ± 4.70 ^b^	ND	212.45 ± 1.21 ^a^
Stigmasterol	144.45 ± 1.20 ^b^	ND	212.90 ± 1.45 ^a^
*β*-Sitosterol	487.41 ± 3.03 ^b^	101.96 ± 1.06 ^c^	625.08 ± 6.30 ^a^
Cycloartenol	43.74 ± 0.60	ND	ND
Brassica sterol	29.88 ± 0.17	ND	ND

The mean ± SD (*n* = 3) is used to express values. Within the same row in the parameter, means with various letters (a, b, and c) differ significantly at *p* < 0.05. ND: not determined. WF1 = watermeal cultivated under farm conditions; WF2 and WF3 = watermeal harvested from natural environments.

**Table 5:** Amino acid composition of watermeal from different growing sites.

Amino Acids (AAs, g/100 g DW)	WF1	WF2	WF3	WHO/FAO/UNU for Adults [41]
Essential amino acids (EAAs)				
Arginine	1.95 ± 0.10 ^b^	2.13 ± 0.05 ^a^	1.32 ± 0.03 ^c^	-
Histidine	0.34 ± 0.01 ^b^	0.24 ± 0.01 ^c^	0.42 ± 0.02 ^a^	1.5
Isoleucine	1.32 ± 0.02 ^a^	1.13 ± 0.03 ^b^	0.92 ± 0.02 ^c^	3.0
Leucine	1.24 ± 0.03 ^a^	1.04 ± 0.04 ^b^	0.87 ± 0.02 ^c^	5.9
Lysine	0.29 ± 0.02 ^b^	0.47 ± 0.01 ^a^	0.46 ± 0.02 ^a^	4.5
Methionine	0.14 ± 0.01 ^b^	0.14 ± 0.01 ^b^	0.18 ± 0.01 ^a^	2.2
Phenylalanine	1.01 ± 0.01 ^c^	1.36 ± 0.03 ^a^	1.18 ± 0.01 ^b^	3.8
Threonine	0.12 ± 0.01 ^b^	0.22 ± 0.02 ^a^	0.14 ± 0.01 ^b^	2.3
Tryptophan	0.45 ± 0.01 ^b^	0.58 ± 0.01 ^a^	0.45 ± 0.01 ^b^	0.6
Valine	1.15 ± 0.04 ^b^	1.03 ± 0.07 ^c^	1.67 ± 0.11 ^a^	3.9
ƩEAAs	8.26 ± 0.13 ^a^	8.25 ± 0.18 ^a^	7.59 ± 0.13 ^b^	-
EAAI ^ns^	0.21 ± 0.01	0.18 ± 0.01	0.20 ± 0.01	-
Non-Essential amino acids (NEAAs)				
Alanine	2.05 ± 010 ^a^	1.17 ± 0.03 ^b^	1.07 ± 0.05 ^c^	
Asparagine	0.36 ± 0.02 ^c^	0.99 ± 0.08 ^a^	0.81 ± 0.05 ^b^	
Aspartic acid	0.51 ± 0.02 ^c^	0.75 ± 0.04 ^a^	0.61 ± 0.01 ^b^	
Cysteine	0.03 ± 0.00 ^c^	0.05 ± 0.00 ^b^	0.07 ± 0.00 ^a^	
Glutamine	0.62 ± 0.03 ^c^	1.08 ± 0.01 ^a^	0.75 ± 0.03 ^b^	
Glutamic acid	0.77 ± 0.03 ^a^	0.49 ± 0.03 ^c^	0.63 ± 0.02 ^b^	
Glycine	0.27 ± 0.01 ^a^	0.17 ± 0.02 ^c^	0.24 ± 0.01 ^b^	
Proline	1.00 ± 0.03 ^a^	0.03 ± 0.00 ^c^	0.34 ± 0.02 ^b^	
Serine	0.19 ± 0.01 ^b^	0.37 ± 0.02 ^a^	0.36 ± 0.01 ^a^	
Tyrosine	1.66 ± 0.03 ^a^	1.45 ± 0.02 ^b^	1.57 ± 0.07 ^a^	
ƩNEAAs	7.51 ± 0.21 ^a^	6.83 ± 0.11 ^b^	6.42 ± 0.15 ^c^	
ƩAAs	15.77 ± 0.23 ^a^	15.08 ± 0.21 ^b^	14.01 ± 0.07 ^c^	
ƩEAAs/ƩAAs (%)	52.37	54.70	54.16	
ƩNEAAs/ƩAAs (%)	47.63	45.29	45.84	

The mean ± SD (*n* = 3) is used to express values. Within the same row in the parameter, means with various letters (a, b, and c) differ significantly at *p* < 0.05. ns is not significantly different at *p* > 0.05. WF1 = watermeal cultivated under farm conditions; WF2 and WF3 = watermeal harvested from natural environments.

## Data Availability

The original contributions presented in this study are included in the article/Supplementary Materials. Further inquiries can be directed to the corresponding author.

## Supplementary Materials

The following supporting information can be downloaded at: https://www.mdpi.com/article/10.3390/foods14101832/s1. Figure S1. GC chromatogram for analysis of phytosterol of watermeal from different growing sites., WF1 = watermeal cultivated under farm conditions; WF2 and WF3 = watermeal harvested from natural environments. Figure S2. LC/MS/MS chromatogram for analysis of amino acid composition in WF1. Figure S3. LC/MS/MS chromatogram for analysis of amino acid composition in WF2. Figure S4. LC/MS/MS chromatogram for analysis of amino acid composition in WF3. Figure S5. GC chromatogram for analysis of fatty acid composition of watermeal from different growing sites., WF1 = watermeal cultivated under farm conditions; WF2 and WF3 = watermeal harvested from natural environments.
