# Supplementary material for: Nutritional Composition and Bioactive Profiles of Farmed and Wild Watermeal (Wolffia globosa)
Source: Foods. 2025 May 21;14(10):1832. doi: 10.3390/foods14101832 (PMC12111385; doi:10.3390/foods14101832)
Supplement: Supplementary file 1 [file foods-14-01832-s001.zip › foods-3646395-supplementary.pdf]

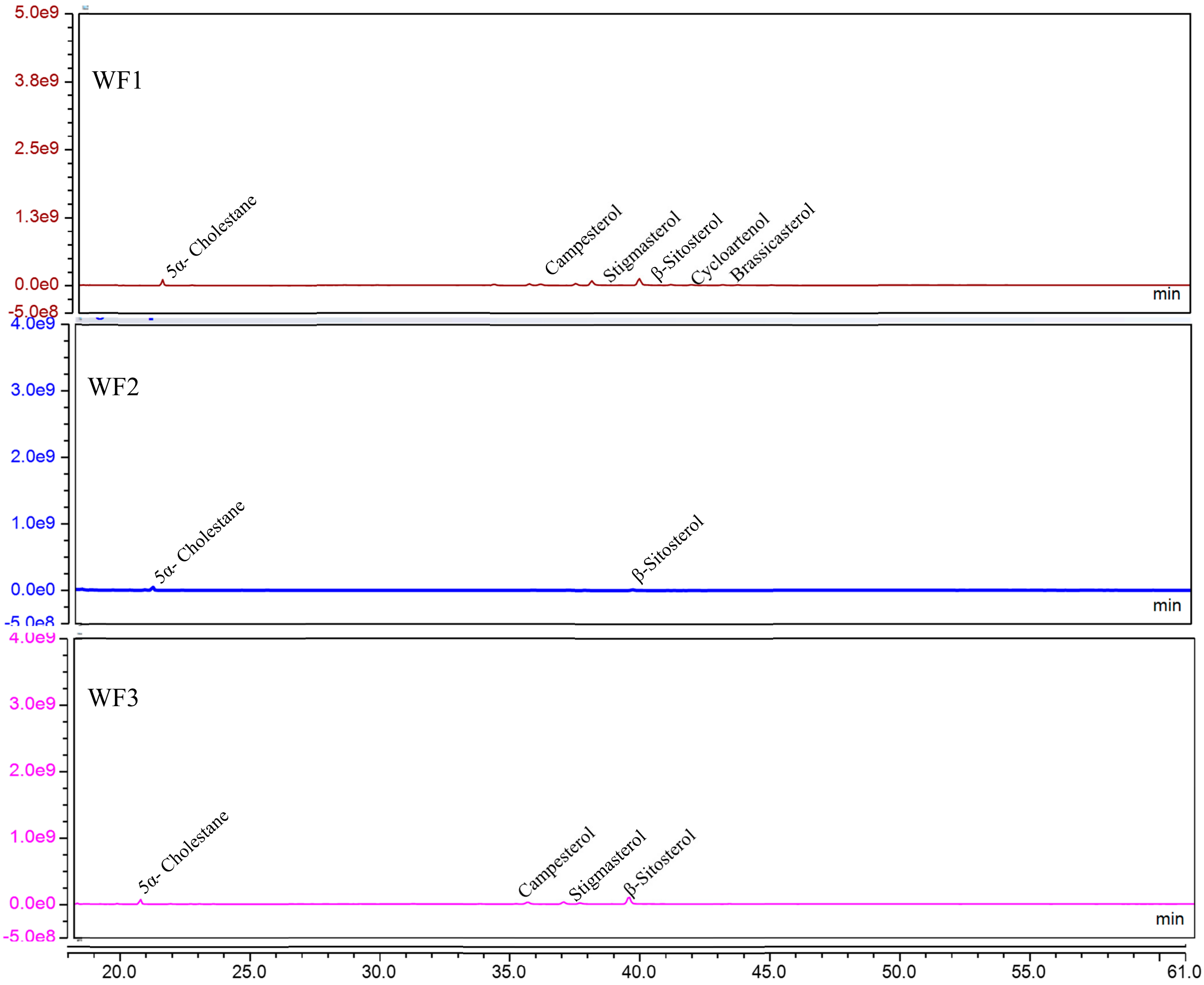


**Figure S1.** GC chromatogram for analysis of phytosterol of watermeal from different growing sites., WF1 = watermeal cultivated under farm conditions; WF2 and WF3 = watermeal harvested from natural environments.

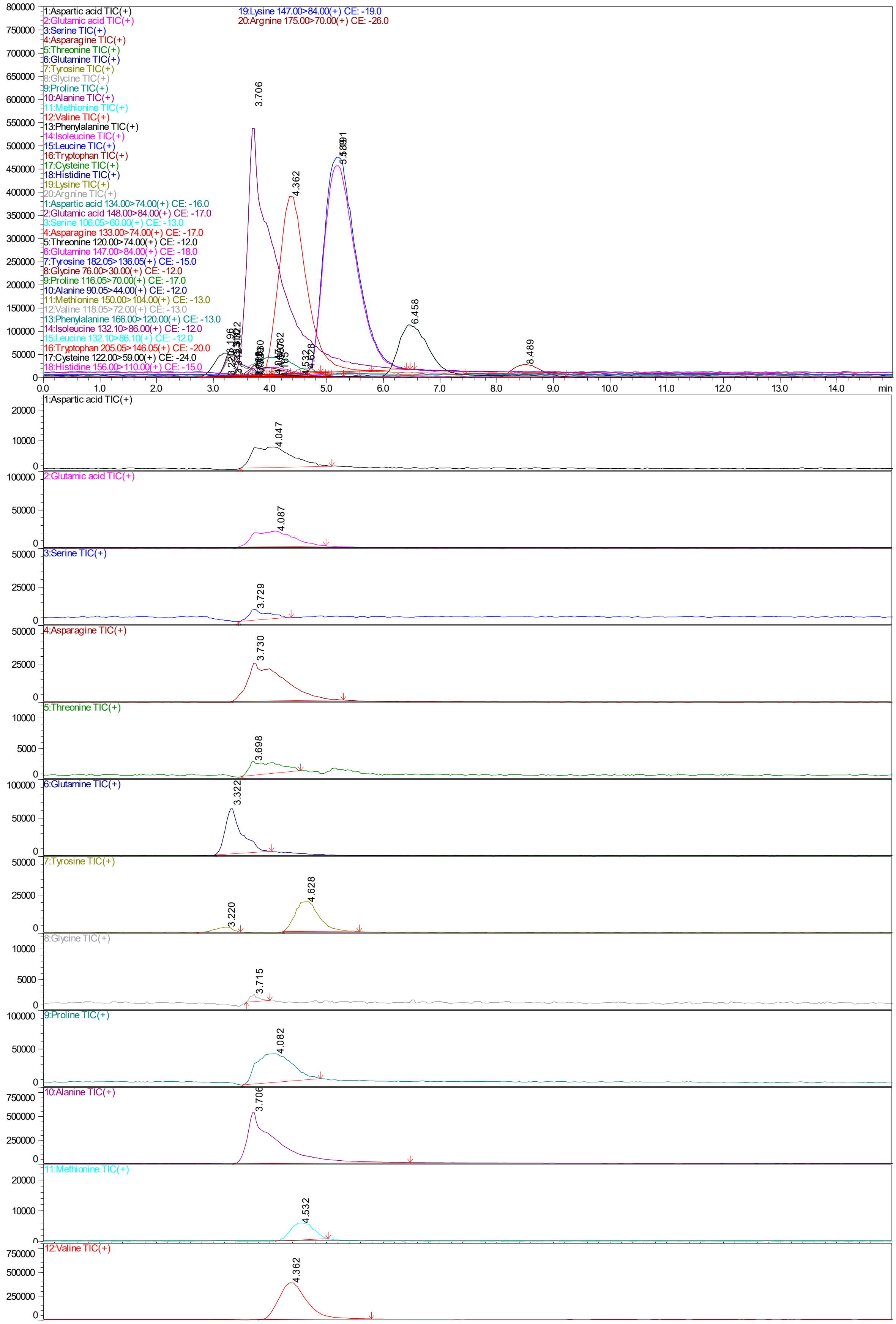
1:Aspartic acid TIC(+)
2:Glutamic acid TIC(+)
3:Serine TIC(+)
4:Asparagine TIC(+)
5:Threonine TIC(+)
6:Glutamine TIC(+)
7:Tyrosine TIC(+)
8:Glycine TIC(+)
9:Proline TIC(+)
10:Alanine TIC(+)
11:Methionine TIC(+)
12:Valine TIC(+)
13:Phenylalanine TIC(+)
14:Isoleucine TIC(+)
15:Leucine TIC(+)
16:Tryptophan TIC(+)
17:Cysteine TIC(+)
18:Histidine TIC(+)
19:Lysine TIC(+)
20:Argnine TIC(+)
1:Aspartic acid 134.00>74.00(+) CE: -16.0
2:Glutamic acid 148.00>84.00(+) CE: -17.0
3:Serine 106.05>60.00(+) CE: -13.0
4:Asparagine 133.00>74.00(+) CE: -17.0
5:Threonine 120.00>74.00(+) CE: -12.0
6:Glutamine 147.00>84.00(+) CE: -18.0
7:Tyrosine 182.05>136.05(+) CE: -15.0
8:Glycine 76.00>30.00(+) CE: -12.0
9:Proline 116.05>70.00(+) CE: -17.0
10:Alanine 90.05>44.00(+) CE: -12.0
11:Methionine 150.00>104.00(+) CE: -13.0
12:Valine 118.05>72.00(+) CE: -13.0
13:Phenylalanine 166.00>120.00(+) CE: -13.0
14:Isoleucine 132.10>86.00(+) CE: -12.0
15:Leucine 132.10>86.10(+) CE: -12.0
16:Tryptophan 205.05>146.05(+) CE: -20.0
17:Cysteine 122.00>59.00(+) CE: -24.0
18:Histidine 156.00>110.00(+) CE: -15.0
19:Lysine 147.00>84.00(+) CE: -19.0
20:Argnine 175.00>70.00(+) CE: -26.0
3.706
4.362
6.458
8.489
min
1:Aspartic acid TIC(+)
4.047
2:Glutamic acid TIC(+)
4.087
3:Serine TIC(+)
3.729
4:Asparagine TIC(+)
3.730
5:Threonine TIC(+)
3.698
6:Glutamine TIC(+)
3.322
7:Tyrosine TIC(+)
3.220
4.628
8:Glycine TIC(+)
3.715
9:Proline TIC(+)
4.082
10:Alanine TIC(+)
3.706
11:Methionine TIC(+)
4.532
12:Valine TIC(+)
4.362

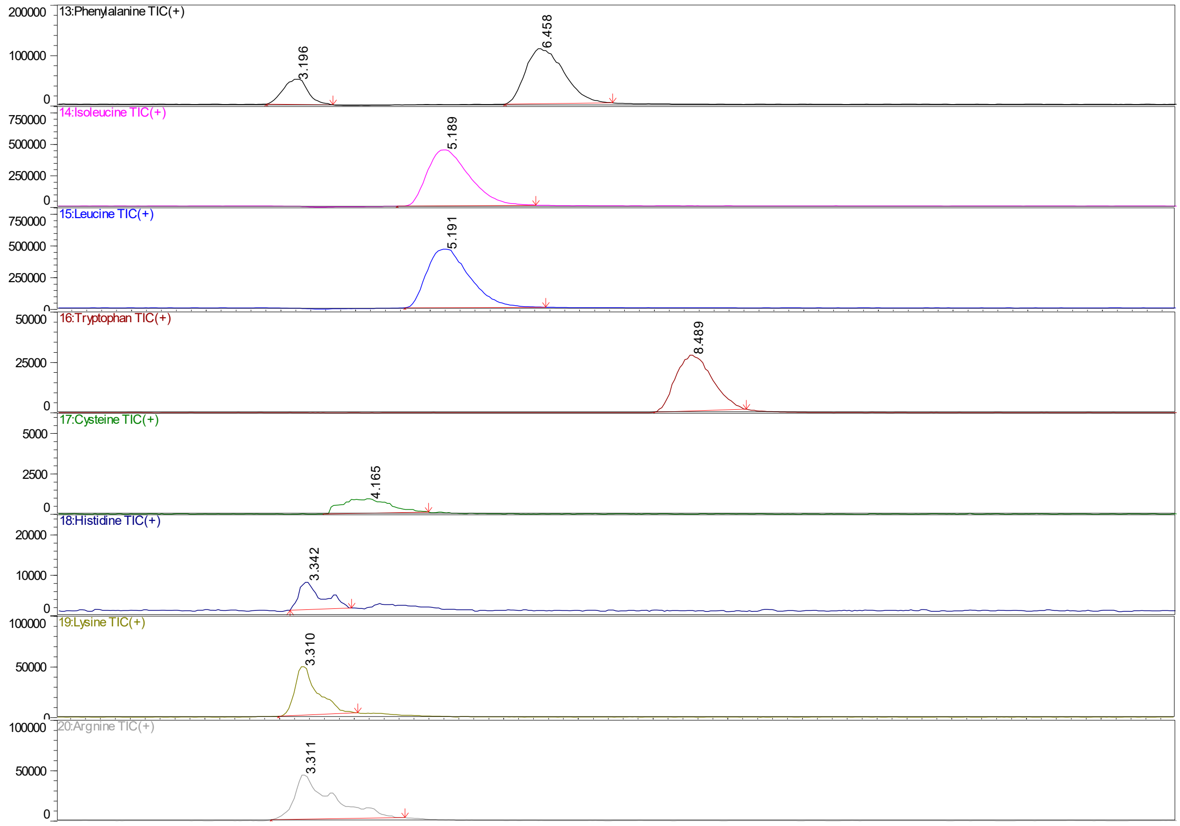


**Figure S2.** LC/MS/MS chromatogram for analysis of amino acid composition in WF1.

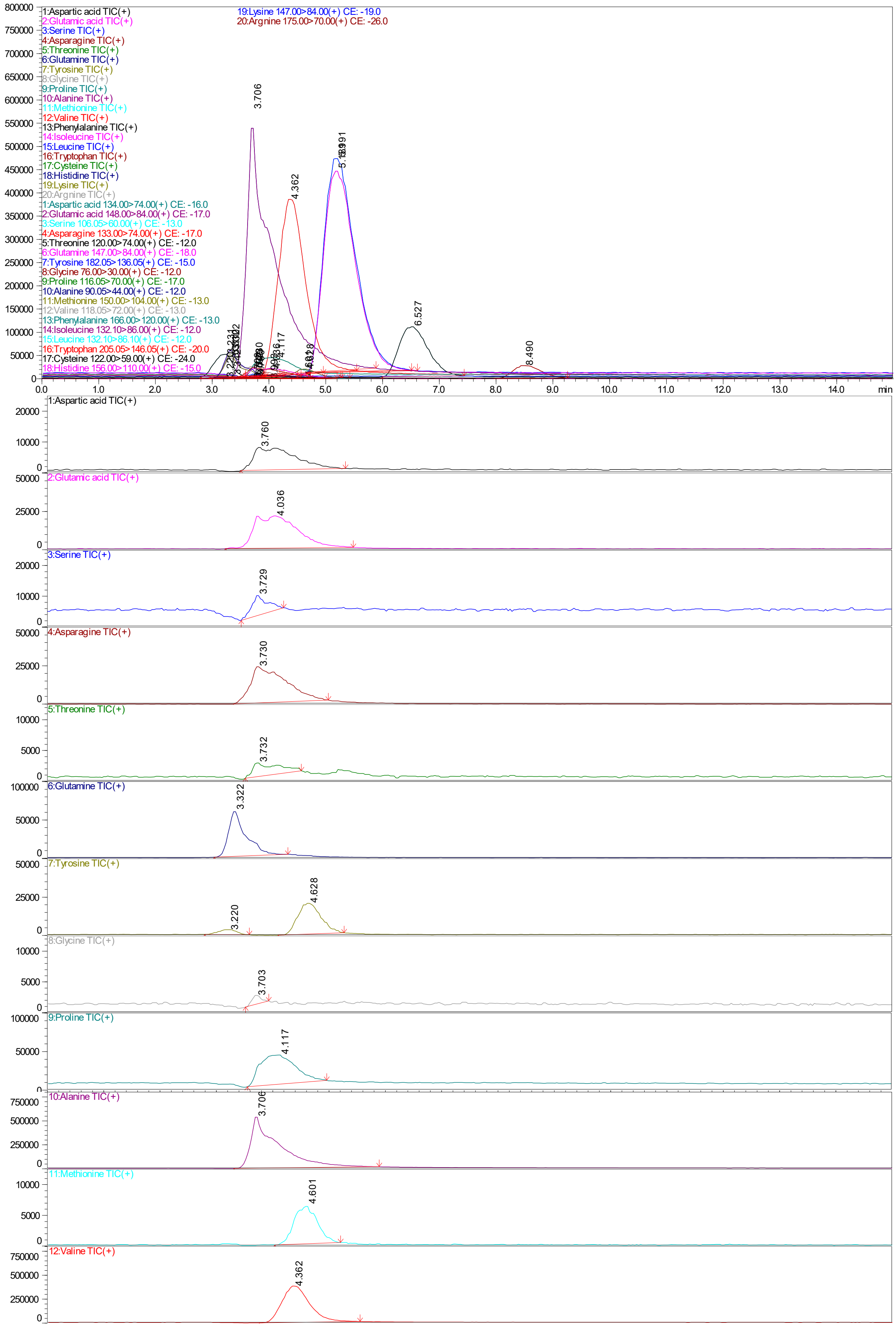

1:Aspartic acid TIC(+)
2:Glutamic acid TIC(+)
3:Serine TIC(+)
4:Asparagine TIC(+)
5:Threonine TIC(+)
6:Glutamine TIC(+)
7:Tyrosine TIC(+)
8:Glycine TIC(+)
9:Proline TIC(+)
10:Alanine TIC(+)
11:Methionine TIC(+)
12:Valine TIC(+)
13:Phenylalanine TIC(+)
14:Isoleucine TIC(+)
15:Leucine TIC(+)
16:Tryptophan TIC(+)
17:Cysteine TIC(+)
18:Histidine TIC(+)
19:Lysine TIC(+)
20:Argnine TIC(+)
1:Aspartic acid 134.00>74.00(+) CE: -16.0
2:Glutamic acid 148.00>84.00(+) CE: -17.0
3:Serine 106.05>60.00(+) CE: -13.0
4:Asparagine 133.00>74.00(+) CE: -17.0
5:Threonine 120.00>74.00(+) CE: -12.0
6:Glutamine 147.00>84.00(+) CE: -18.0
7:Tyrosine 182.05>136.05(+) CE: -15.0
8:Glycine 76.00>30.00(+) CE: -12.0
9:Proline 116.05>70.00(+) CE: -17.0
10:Alanine 90.05>44.00(+) CE: -12.0
11:Methionine 150.00>104.00(+) CE: -13.0
12:Valine 118.05>72.00(+) CE: -13.0
13:Phenylalanine 166.00>120.00(+) CE: -13.0
14:Isoleucine 132.10>86.00(+) CE: -12.0
15:Leucine 132.10>86.10(+) CE: -12.0
16:Tryptophan 205.05>146.05(+) CE: -20.0
17:Cysteine 122.00>59.00(+) CE: -24.0
18:Histidine 156.00>110.00(+) CE: -15.0
19:Lysine 147.00>84.00(+) CE: -19.0
20:Argnine 175.00>70.00(+) CE: -26.0
3.706
4.362
6.527
8.490
min
1:Aspartic acid TIC(+)
3.760
2:Glutamic acid TIC(+)
4.036
3:Serine TIC(+)
3.729
4:Asparagine TIC(+)
3.730
5:Threonine TIC(+)
3.732
6:Glutamine TIC(+)
3.322
7:Tyrosine TIC(+)
3.220
4.628
8:Glycine TIC(+)
3.703
9:Proline TIC(+)
4.117
10:Alanine TIC(+)
3.706
11:Methionine TIC(+)
4.601
12:Valine TIC(+)
4.362

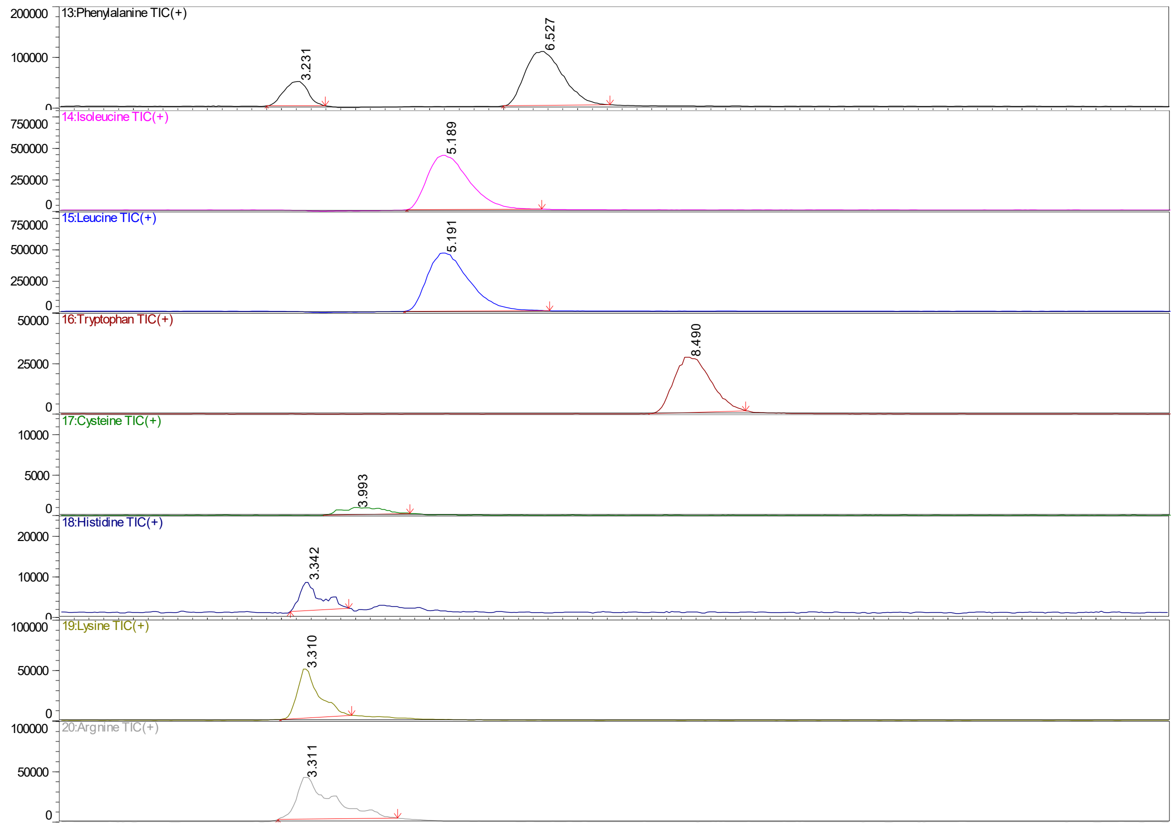


**Figure S3.** LC/MS/MS chromatogram for analysis of amino acid composition in WF2.

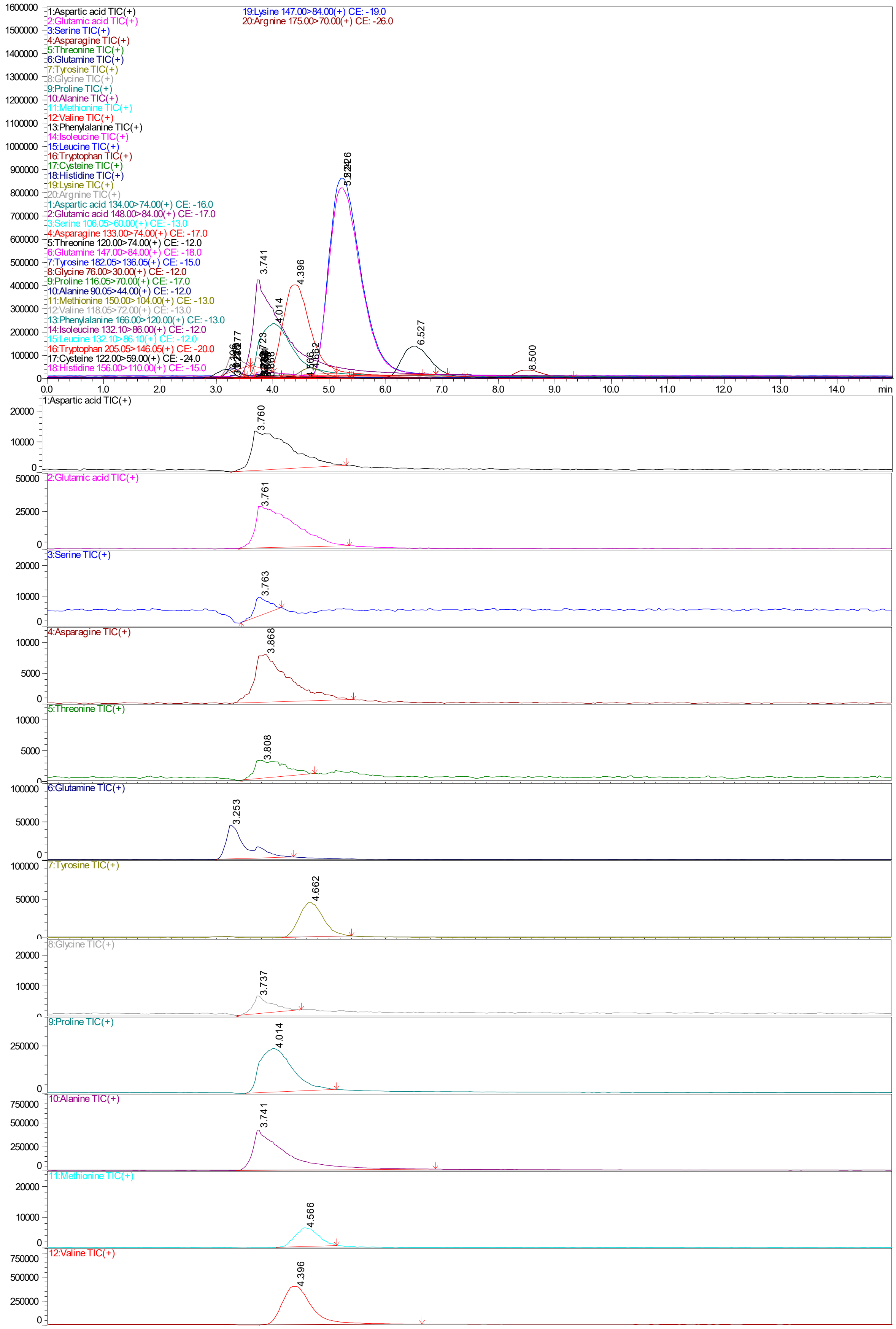
1:Aspartic acid TIC(+)
2:Glutamic acid TIC(+)
3:Serine TIC(+)
4:Asparagine TIC(+)
5:Threonine TIC(+)
6:Glutamine TIC(+)
7:Tyrosine TIC(+)
8:Glycine TIC(+)
9:Proline TIC(+)
10:Alanine TIC(+)
11:Methionine TIC(+)
12:Valine TIC(+)
13:Phenylalanine TIC(+)
14:Isoleucine TIC(+)
15:Leucine TIC(+)
16:Tryptophan TIC(+)
17:Cysteine TIC(+)
18:Histidine TIC(+)
19:Lysine TIC(+)
20:Argnine TIC(+)
1:Aspartic acid 134.00>74.00(+) CE: -16.0
2:Glutamic acid 148.00>84.00(+) CE: -17.0
3:Serine 106.05>60.00(+) CE: -13.0
4:Asparagine 133.00>74.00(+) CE: -17.0
5:Threonine 120.00>74.00(+) CE: -12.0
6:Glutamine 147.00>84.00(+) CE: -18.0
7:Tyrosine 182.05>136.05(+) CE: -15.0
8:Glycine 76.00>30.00(+) CE: -12.0
9:Proline 116.05>70.00(+) CE: -17.0
10:Alanine 90.05>44.00(+) CE: -12.0
11:Methionine 150.00>104.00(+) CE: -13.0
12:Valine 118.05>72.00(+) CE: -13.0
13:Phenylalanine 166.00>120.00(+) CE: -13.0
14:Isoleucine 132.10>86.00(+) CE: -12.0
15:Leucine 132.10>86.10(+) CE: -12.0
16:Tryptophan 205.05>146.05(+) CE: -20.0
17:Cysteine 122.00>59.00(+) CE: -24.0
18:Histidine 156.00>110.00(+) CE: -15.0
19:Lysine 147.00>84.00(+) CE: -19.0
20:Argnine 175.00>70.00(+) CE: -26.0
3.741
4.396
4.014
6.527
8.500
min
1:Aspartic acid TIC(+)
3.760
2:Glutamic acid TIC(+)
3.761
3:Serine TIC(+)
3.763
4:Asparagine TIC(+)
3.868
5:Threonine TIC(+)
3.808
6:Glutamine TIC(+)
3.253
7:Tyrosine TIC(+)
4.662
8:Glycine TIC(+)
3.737
9:Proline TIC(+)
4.014
10:Alanine TIC(+)
3.741
11:Methionine TIC(+)
4.566
12:Valine TIC(+)
4.396

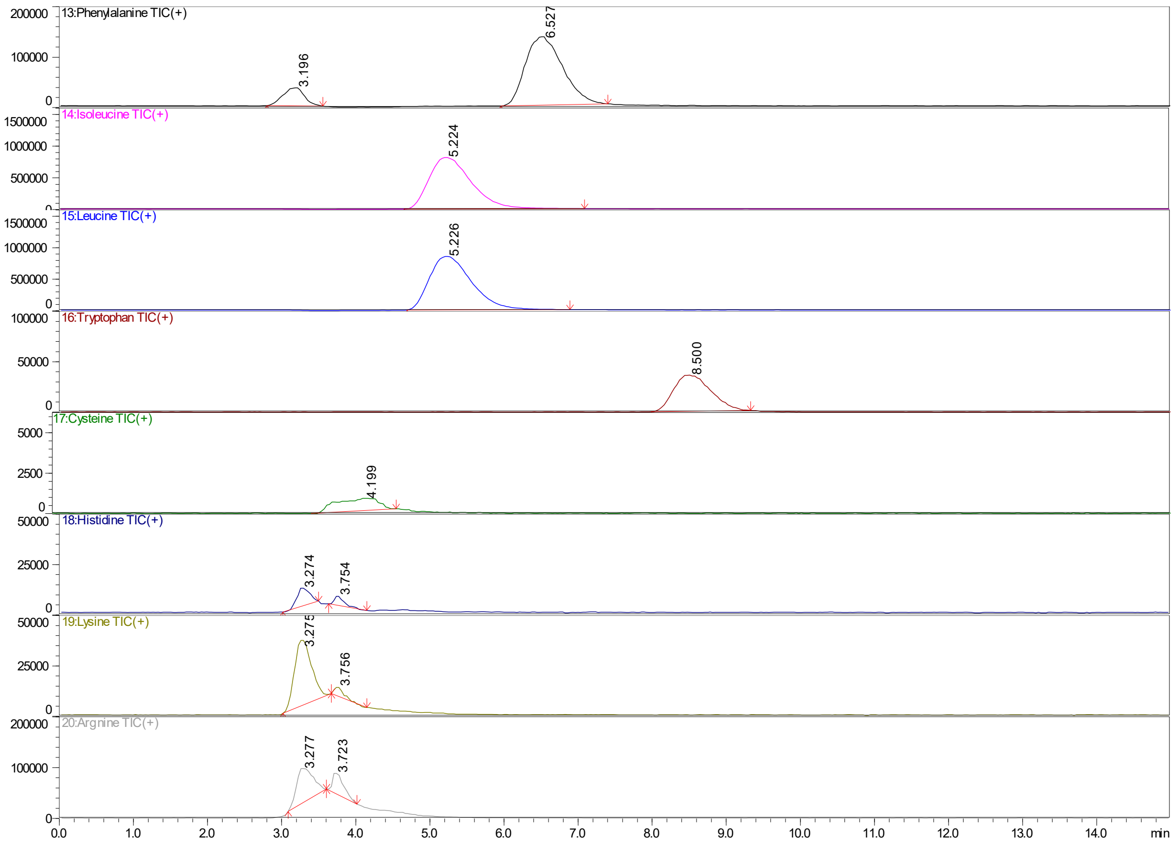


**Figure S4.** LC/MS/MS chromatogram for analysis of amino acid composition in WF3.

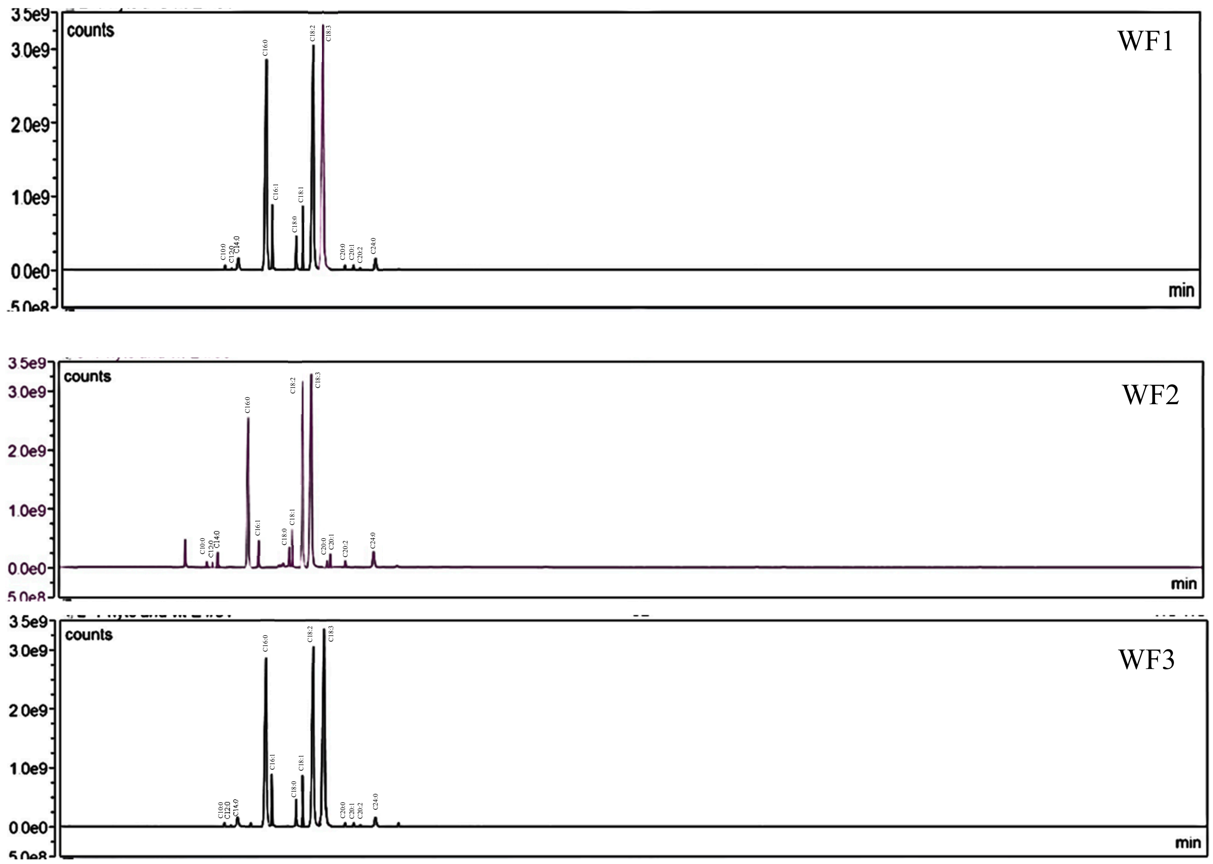


**Figure S5.** GC chromatogram for analysis of fatty acid composition of watermeal from different growing sites., WF1 = watermeal cultivated under farm conditions; WF2 and WF3 = watermeal harvested from natural environments.
